# 

**Published:** 1894-06-23

**Authors:** 


CONTENTS.

Germs not Pathological - 243
The History and Capabilities of Herbal Simples. By W. T. Fernie, M.D —Hemlock and Henbane - 244
Some Further Notes on the Hydrides. By Sir Benjamin Ward Richardson, M.D , F.R.S. - 245
The Plagues of the Bible - 246
Modern Medico Psychology and Psychiatry. By T. S. Clouston, M.D.—The Forms and Classification of Mental Diseases - 247
Studies in Applied Sociology. XI.—How Workers Live - 248
Annotations.—Can Doctors Prevent Insanity?—Unconscious Self-Poisoning.—Medical Protection - 249
Notes and News - 250
Scraps and Gleanings - 262
Continental Notes from a Holiday Correspondent.—Paris 263
Medical Progress and Hospital Clinics.—Remarks on the Gravity of any Discharge from the Ear - 251
Some Simple Uses of Carbolic Acid. II. - 252
Medical Progress.—Typhoid Fever - 253
Progress in Surgery.—Hernia - 255
Appendicitis - 256
New Drugs and Preparations - 257
New Appliances and Things Medical - 257
Editor's Letter-Box.—The Birmingham Hospital Saturday Fund 258
The Institutional Workshop:
Hospital Finance.—VIII. A Provident Scheme - 259
St. Thomas's Hospital - 260
Hospital Festivals and Meetings—Construction Notes - 262

EXTRA SUPPLEMENT.—THE NURSING MIRROR.

News from the Nursing World.—Our Princess—Trained and Untrained—Complaints Discouraged—Help Urgently Needed—An Inquiry Courted—Pet Economies—After Five Years—Prince Alfred Hospital, Sydney—Another Brave Nurse—The Nurses' Co-operation—Short Items - cxi
On General Nursing.—XVII. Latent and Chronic Rheumatism cxiii
The Mechanism of Movement —IV. - cxiv
Appointments—Notes and Queries - cxiv
Character in the Insane - cxv
On Private Nursing in Connection with Hospitals - cxvi
Workhouse Infirmary—Wants and Workers - cxvi
The Children's Country Holidays' Fund - cxvii
Nursing in Germany - cxvii
Notes from Melbourne—For Reading to the Sick - cxviii
An Emergency - cxviii
The Muses Looking Glass - cxix
Everybody's Opinion - cxx
Where to Go - cxx

